# Borderline personality disorder in East Asian adolescents: a comprehensive review of research on assessment, etiology, and treatment

**DOI:** 10.3389/fpsyt.2026.1805312

**Published:** 2026-05-08

**Authors:** Huijuan Zhang, Yifan Lyu, Ansi Qi, Jing Wen, Lanlan Wang

**Affiliations:** 1 Department of Psychological Medicine, Shanghai General Hospital, Shanghai Jiao Tong University School of Medicine, Shanghai, China; 2 Shanghai Mental Health Center, Shanghai Jiao Tong University School of Medicine, Shanghai, China

**Keywords:** adolescents, borderline personality disorder, East Asia, etiology, treatment

## Abstract

Borderline personality disorder (BPD) is a severe mental disorder marked by instability in affect, impulse control, interpersonal relationships, and behaviors. Its symptoms typically appear during adolescence and may predict adverse long-term outcomes. In East Asia, research focusing on this population is growing. This review aims to comprehensively summarize these findings to provide an overall profile of adolescents with BPD in East Asia. Studies show the prevalence of BPD among East Asian adolescents ranges from 2% to 14%, depending on the assessment instruments and the study population. Several diagnostic instruments have been developed and validated accordingly. Childhood trauma, especially emotional abuse, constitutes a significant risk factor for BPD. Other contributing factors include ineffective family environments, maladaptive parenting practices, peer aggression, and urban-rural disparities. Emotional dysregulation is a core symptom, along with interpersonal and identity disturbances. Self-injury and suicidal behaviors may show certain different patterns, and BPD features frequently overlap with other psychiatric disorders, especially when comorbid. Dialectical Behavior Therapy (DBT) and Mentalization-Based Treatment (MBT) are increasingly implemented, though their adoption varies across East Asia, and high-quality intervention research remains limited. Future studies should consider the East Asian cultural context, develop early identification methods and intervention strategies, integrate family and school systems, and explore individualized treatment pathways.

## 1 Introduction

BPD is characterized by a pervasive pattern of instability of interpersonal relationships, self-image, and affect, and marked impulsivity. BPD has been recognized as a condition with significant public health implications, mainly because it contributes to functional impairment, psychiatric comorbidity, and elevated risk of self-harm and premature mortality (1). Increasing evidence indicates that core borderline traits have an onset in adolescence or early adulthood. Adolescent BPD is not a transient phase-limited disturbance, but rather can predict persistent psychosocial dysfunction, recurrent self-injurious behaviors, and adverse adult outcomes (1). The detection and treatment of subsyndromal borderline pathology or categorical BPD are now recognized as playing an important role in mental health preventative treatment (2). In line with this new development, new methods are constantly being developed in the field of diagnostics for assessing BPD in younger individuals.

Both DSM-5 and ICD-11 allow for the diagnosis of BPD in adolescents, emphasizing the importance of identifying persistent and pervasive symptomatology. The DSM-5 requires at least one year of symptoms meeting five or more BPD criteria in adolescents. Its alternative dimensional model further requires moderate impairments in personality functioning across domains such as identity and empathy, along with specific pathological traits (3). The ICD-11 focuses on grading severity (“mild” to “severe”) across five trait domains, and its borderline pattern specifier can be applied when an individual meets five of the nine adapted DSM-5 BPD criteria. As for medication, there is no consistent evidence that any psychiatric drugs really help with the core symptoms in BPD adults (4). Therefore, psychological therapy, as the cornerstone of intervention - especially during the early developmental stage, when neuroplasticity and the social psychological environment may offer unique treatment opportunities - is particularly important.

While there has been a significant increase in the study of adolescent BPD, most of these studies are based on Western samples. It is unclear if these findings apply to East Asia, because factors such as collectivist values, family relationships, academic pressure, and mental health stigma really affect how BPD shows up, gets noticed, and is treated. In East Asia, research on teenage BPD has drawn more attention. This has been in both the community and the clinical setting. Therefore, this review aims to synthesize the current evidence on adolescent BPD in East Asia, encompassing prevalence, diagnostic assessment, clinical features, risk and protective factors, and treatment approaches. Furthermore, it highlights key similarities and differences with Western research and outlines culturally informed directions for future research and clinical practice.

## 2 Prevalence

Reported prevalence rates of BPD among East Asian adolescents vary considerably across studies, influenced by assessment tools, sampling strategies, and cultural-regional contexts. The key findings are presented in **Table 1**. In a study from Hong Kong, a total of 4,110 high school students completed the McLean Screening Instrument for Borderline Personality Disorder (MSI-BPD) and other measures assessing various BPD traits twice over one year. While the MSI-BPD alone yielded prevalence estimates of 5–7%, a stringent simulated diagnostic procedure estimated the prevalence rate of BPD to be 2% in this population (5). In Taiwan, a sample of 2,067 students completed the Chinese Personality Disorder Inventory–BPD Criteria (CPDI-BPD) questionnaire. Results indicated that approximately 7% of participants exhibited borderline personality traits (6). In mainland China, two public school studies using different instruments reported higher rates. One study involving 1,147 adolescents administered the Self−Report Dissociative Disorders Interview Schedule for BPD (SR−DDIS−BPD) and found that 9.9% endorsed five or more BPD features (7). Another cross−sectional online survey of 2,664 senior high school students, using self−report measures PDQ-4, reported a prevalence of 12% for borderline personality traits (8). A study of 4,866 middle school students in southwest China using the MSI-BPD reported a prevalence of 14% (9).

**Table 1:** Assessment Tools and Epidemiological Surveys of Adolescent BPD in East Asia.

Author/Published year	Region	Subjects	Age (years old)	Sample size	Scale	Reliability and validity	Assessment methods	Prevalence
Leung SW. et al., 2009 (5)	Hong Kong	high school students	12-20	4110	MSI-BPD	Internal consistency was 0.74, and retest reliability was 0.72	self-report	2%*
Liu M. et al., 2011 (6)	Taiwan	high school students	NA	2067	CPDI-BPD Criteria	NA	self-report	7%
Yuan GF. et al., 2025 (7)	Mainland China	high school students	16.40 ± 0.81	1147	SR-DDIS-BPD	NA	self-report	9.90%
Zhong Y. et al., 2024 (8)	Mainland China	high school students	14-18	2664	PDQ-4	Cronbach’s α was 0.678	self-report	12%
Wang Q. et al., 2024 (9)	Mainland China	middle school students	10-18	4866	MSI-BPD	Cronbach’s α was 0.85	self-report	14%
Li J.et al., 2018 (10)	Mainland China	high school students	15-19	446	BPFS-C	Cronbach’s α was 0.80	self-report	NA
Liu P., 2019 (11)	Mainland China	high school and primary school students	9-18	964	BPFS-C	Cronbach’s α of the scale was 0.853; retest reliability was 0.824	self-report	NA
Zhuo C. et al., 2022 (12)	Mainland China	BPD adolescent inpatients	10-17	120	BPFSC-SF	Cronbach’s α of the scale was 0.854	self-report	NA
Wang J., 2024 (13)	Mainland China	high school students	12-18	1126	BPFSC-11	Cronbach’s α of the scale was 0.908, and retest reliability was 0.807	self-report	NA
Shen JE. et al., 2023 (16)	Taiwan	BPD adolescent inpatients	12-18	279	BSL-23	Cronbach’s α of the scale was 0.96; retest reliability was 0.936	self-report	NA

BPD, Borderline Personality Disorder; MSI-BPD, McLean Screening Instrument for Borderline Personality Disorder; CPDI-BPD Criteria, Chinese Personality Disorder Inventory - BPD Criteria; SR-DDIS-BPD, Self-Report Dissociative Disorders Interview Schedule for Borderline Personality Disorder; PDQ-4, Personality Diagnostic Questionnaire-4; BPFS-C, Borderline Personality Features Scale for Children; BPFSC-SF, Borderline Personality Features Scale for Children-Short Form; BPFSC-11, Borderline Personality Features Scale for Children-11; BSL-23, Borderline Symptom List-23.

*the MSI-BPD alone yielded prevalence estimates of 5–7%, while a stringent simulated diagnostic procedure estimated the prevalence at 2%.

Self-report instruments are more frequently utilized in current research on BPD adolescents in East Asia. The reliability, validity, and cross-cultural applicability of several instruments for this population have been established. As shown in **Table 1**, the Chinese version of the Borderline Personality Feature Scale for Children–Short Form (BPFSC-SF) is currently the most widely used instrument in research in China. Multiple studies have confirmed its good internal consistency and high test−retest reliability (10, 11). In a study involving 120 Chinese adolescent BPD patients, the scale demonstrated a Cronbach’s α of 0.854 and ICC of 0.937 (12). Another study, which included a sample of 1,126 middle school students, validated the simplified version of the scale, the BPFSC-11. This simplified version demonstrated an internal consistency α of 0.908 and a test-retest reliability of 0.807 (13). Those results are comparable to those reported in American (α=0.85) (14) and Italian (α=0.78) (15) samples.

The reliability and validity of several other self-report instruments have also been validated, including Borderline Symptom List-23 (BSL-23) (16), the Chinese version of the McLean Screening Instrument for BPD (MSI-BPD) (5), the Self-Report Dissociative Disorders Interview Schedule for Borderline Personality Disorder (SR-DDIS-BPD) (17), and the Chinese revision of the Personality Diagnostic Questionnaire-4 (PDQ-4) (18). Although some semi-structured interviews, such as the Diagnostic Interview for Borderline Personality Disorder-Revised (DIB-R) and Structured Clinical Interview for DSM-IV Axis II Personality Disorders (SCID-II), have been validated and have demonstrated good consistency, reliability, and validity in adults (19–21), there is still a lack of evidence on Asian adolescents. Besides, some assessments that have been applied internationally for adolescent BPD but have not yet been localized or widely adopted in Asia, especially the application of parent-report instruments and semi-structured interviews, need to be validated (22).

These prevalence estimates, ranging from 2% to 14%, highlight considerable variability likely stemming from methodological differences in sampling and assessment. Notably, research on adolescent BPD in East Asia has predominantly utilized school-based or community samples. This contrasts with Western studies, which frequently incorporate data from both community (15, 23) and clinical populations (24–26). The reliance on school-based sampling in East Asia can be attributed to several interrelated factors: clinical recruitment barriers due to pervasive stigma, the expansion of school-based mental health screening programs (27), and diagnostic conventions that historically restrict personality disorder diagnoses in adolescents in clinical settings. In contrast, a recent large-scale study in Western countries has shown that the prevalence rate of BPD is approximately 3% among the general adolescent population, 11% among adolescents in clinical settings, and 78% among suicidal adolescents referred to emergency departments (22). Future cross-cultural comparisons should employ standardized assessment protocols and comparable sampling strategies to distinguish genuine cultural differences from methodological artifacts.

## 3 Etiology and relevant factors

### 3.1 Sociodemographic factors

Several sociodemographic factors are related to Borderline personality features (BPF) in Asian adolescents. At the individual level, age and gender are important factors. Research shows a complex link between age and BPF, with BPF detection rates increasing continuously across childhood and adolescence (11): among adolescents aged 13-17, older age significantly predicts higher BPF scores, with those at the age of 16–17 demonstrating the highest levels (13). However, in samples of high school students, BPD traits are more frequently observed in freshmen and sophomores compared to juniors (28), suggesting a potential peak in mid-adolescence followed by decline. This may be indicative of both the accumulated effects of adverse experiences during early-to-mid adolescence and normative personality maturation in later adolescence. With regard to sex, females show significantly higher BPF detection rates in comparison to their male counterparts, although this difference primarily manifests in the high school years, rather than earlier stages in their development (11).

In the family context, adolescents from single-parent households had significantly higher BPD traits than those from intact families (28), and parental marital instability is also associated with high BPF scores (13). The aforementioned results suggest that the disruption of the stability of family structure might weaken the stable care-providing environment for healthy personality growth. Additionally, it was mentioned that non-singleton status was also a risk factor for BPF (13). Given the recent one-child policy relaxation in China, especially in multi-child families, adolescents might experience unequal allocation of parental resources and less individual attention from the caregivers.

At a societal level, lower perceived social status was found to be associated with high borderline traits (28). Reduced social status at the school level may be a manifestation of peer and teacher relationship difficulties, as well as experiences of marginalization, whereas lower societal status tends to represent a family’s socioeconomic disadvantage and limited access to resources. In addition, residing in rural areas was associated with higher ratings of BPF compared to those residing in urban areas (13). This rural-urban disparity is very prominent in the current era of China, in which the massive development in cities has created significant disparity across multiple areas. In addition, other factors that may expose the adolescents to a higher risk are parental labor migration and prolonged separation from caregivers.

### 3.2 Childhood trauma and maltreatment

East Asian countries have been known to show high rates of childhood maltreatment among adolescents with BPF, and in their experience, emotional abuse and neglect are particularly prevalent (7, 29, 30). The significant positive association between childhood trauma and BPF has consistently been documented across many cross-sectional studies (8, 31). Moreover, Liu (11) also found that maltreatment experience is an independent factor of BPF using a logistic regression analysis. Longitudinal evidence supports this as well: Yang (32) followed 1,648 adolescents at three time points for one year, revealing that developmental trajectories of childhood maltreatment are positively associated with BPF trajectories.

Research investigating the underlying psychological mechanism has also explored this relationship. Childhood trauma may contribute to the development of BPF, in part, by impairing self-related psychological resources. Self-esteem partially mediates the relationship between emotional abuse and BPF (18), whilst psychological adjustment capacity has been considered a protective factor as well (31) due to its partial mediating role between childhood maltreatment and BPF. Similarly, resilience could also serve as a mediating factor in the pathway from childhood abuse to BPF, showcasing that adolescents’ positive adaptation in the face of adverse circumstances may weaken the negative impact of early maltreatment on personality growth (18). Neuroimaging studies also found potential neural correlates of this relationship, as BPD adolescents showed altered activity in the insula (30), which showed a significantly positive correlation with childhood maltreatment.

In particular, converging evidence in East Asian literature reveals the specific salience of emotional forms of maltreatment. In a clinical study from Shanghai that was designed to compare 178 BPD patients with matched controls with other personality disorders or no personality pathology, emotional maltreatment, which included the two subcategories named emotional abuse and emotional neglect, was found to be the most powerful predicting factor of BPD diagnosis (33). The pattern of findings was further confirmed by Xie and coworkers (18), who reported that while the three types of abuse (emotional, physical and sexual) were each independently associated with BPF, when considered simultaneously, only emotional abuse continued to show significant predictive power. Research on clinical (33) and community (18) samples concluded with convergence that, while there are possible contributions of multiple forms of maltreatment, emotional abuse plays the strongest and most robust role in the development of BPD in East Asian populations.

### 3.3 Parenting practices and family environments

In addition, parenting practices and family environments are also strong factors influencing adolescents’ development of BPF. Warm, emotionally supportive parenting may exert protective effects on the development of borderline pathology (34), whereas maladaptive parenting patterns (rejection, overprotection, and anxious rearing) were found to be positively associated with BPF, with rejection having the strongest association (r=0.423, p<.001) (34). Furthermore, binary logistic regression analyses confirmed that poor parenting practices and poor attachment relationships with parents acted as independent risk factors for BPF (11), suggesting that adverse parenting contributes to the development of borderline pathology in the youth populations in East Asian countries. In addition to these direct associations, exploration of the mediating mechanisms has revealed the pathway of how adverse parenting ultimately led to the development of BPF. Mentalization or reflective functioning, namely the ability to understand one’s own and others’ mental states, appeared to be a significant protective factor. Guo et al. (34) discovered reflective functioning was strongly negatively correlated with BPF (r=-0.666, p <.001). Maladaptive parenting practices hindered the adolescents from properly establishing their reflective functioning, consequently weakening their ability to regulate their emotions, and finally leading to the development of BPF (34).

Invalidating family environments, characterized by parental responses that dismiss, punish, or fail to acknowledge children’s emotional experiences, have been identified as a key factor in the development of BPF, supporting Linehan’s biosocial developmental theory of BPD (35). Another study with 483 Chinese adolescents indicated that group differences in impulse control between high and low BPF adolescents ceased to be significant after statistically controlling for the invalidating family environment (36). These findings suggest that the invalidating family environment is an important explanatory variable relating the family context to BPD core impairment rather than merely a correlate.

### 3.4 Peer victimization and peer attachment

Beyond the family context, the broader interpersonal environment in which adolescents are embedded also plays a role in BPF development. Research has identified peer-related factors as important contributors to BPF development among Chinese adolescents. A longitudinal study of junior middle school students found that increases in peer victimization over time were directly associated with increases in borderline features, and the quality of peer attachment was identified as another factor in BPF development (32). Peer attachment was proven to be an independent factor for BPF in another Chinese study (11).

## 4 Clinical symptoms

### 4.1 Affective dysregulation

Affective dysregulation may represent a core feature in East Asian adolescents with BPD. A community-based study in China identified a strong positive correlation between emotional instability and BPD traits, with coefficients reaching 0.699 (34). Notably, adolescents from single-parent families have been found to score higher on both BPD features and emotional regulation difficulties, suggesting that family structure may interact with emotional vulnerability (34). Besides, a study in a western city in China found that emotional regulation difficulties—together with interpersonal sensitivity, paranoid ideation, loneliness, and deliberate self-harm—collectively accounted for 55.7% of the variance in borderline traits (13).

Deficits in emotional processing further complicate the pathway from BPD traits to non-suicidal self-injury (NSSI). In a sample of 1,192 depressed Chinese adolescents, emotional dysregulation was found to significantly mediate the relationship between BPD features and NSSI (37). Alexithymia is defined by difficulties in identifying and describing emotions. A study involving 1,779 psychiatric inpatient adolescents showed that alexithymia is an important mediating factor between NSSI severity and emotional regulation impairment (38). These findings collectively emphasize that effective treatment should not only focus on the intensity of emotions but also enhance individuals’ ability to identify and express them.

### 4.2 Non-suicidal self-injury and suicidal behaviors

Self-injurious behaviors are highly prevalent among East Asian adolescents with BPF. Studies in Western adolescents indicate a lifetime prevalence of NSSI exceeding 90% among clinical BPD samples (39). A study in Taiwan students found that 9.7% reported engaging in self-injurious behavior (6). Adolescents with BPF demonstrated a 22-fold increased relative risk of self-injury compared to those without such traits (6). These adolescents also used more varied methods of self-harm and held more negative attitudes toward life, which has been associated with more severe clinical presentations, including higher emotion dysregulation and greater self-criticism (40).

Research on NSSI methods among East Asian adolescents with BPD remains limited. The study from the Taiwan region found that cutting was the most common method (44.4%), followed by punching walls (35.6%), and carving on skin (28.9%) (6). These rates appear notably lower than those reported in Western adolescent clinical samples, where cutting prevalence typically ranges from 80-100% (24, 25, 39, 41), and other methods, such as severe scratching (57.7-72%) and burning (38.5-40%), also show higher prevalence (24, 25, 41). However, these cross-cultural comparisons are limited by methodological heterogeneity. The scarcity of data on NSSI methods in East Asian adolescent BPD undermines its representativeness. Second, critical disparities exist in sampling (community versus clinical inpatient) and assessment (self-report versus structured interviews), confounding rates and severity estimates (25, 26, 41).

Longitudinal research revealed the trajectories of how various factors contributed to self-harm behaviors. In line with the findings in Western adolescents (42), a two-year study of Hong Kong secondary school students found that affective instability, interpersonal disturbances, and behavioral impulsivity each predicted the presence and initiation of NSSI (43). However, only behavioral impulsivity emerged as a unique predictor of repeated NSSI, while lower emotional instability correlated with NSSI cessation (43). This suggests that while emotional pain may trigger self-harm, its chronicity may be explained by trait-like impulsivity. A targeted intervention for impulsivity may prevent chronic self-harm.

### 4.3 Interpersonal instability, identity disturbance, and chronic emptiness

While affective dysregulation and impulsivity are readily identifiable, BPD’s core disturbances in interpersonal functioning, self-concept, and cognition are less visible in East Asian adolescents—yet equally central to the disorder. The symptom profile thus reflects both cultural variations and these methodological limitations. A recent large-scale network analysis in Chinese adolescents revealed that identity disturbance and affective instability emerged as the most central BPF in this sample (9). Critically, “unstable relationships” and “abandonment avoidance” exhibited strong connectivity, confirming that interpersonal dysfunction is core to BPD in this population. Furthermore, chronic emptiness emerged as highly central, suggesting profound cultural influences (9). Compared to Westerners, Chinese adolescents face more parental control and academic overemphasis, especially in an invalidating family. As a result, children have limited time and opportunities to explore personal interests and then develop a sense of meaning, rendering them prone to existential purposelessness rather than affective void (44). Maladaptive parenting also impairs reflective functioning, suggesting a mechanism whereby family invalidation disrupts interpersonal understanding, thereby leading to increasing emptiness (34). Besides, a high proportion of dissociation was found in Chinese adolescents, which raises questions about cultural interpretation: “feeling unreal” may reflect mental resistance to reality rather than derealization (9).

### 4.4 Comorbidity and differential diagnosis

Comorbid conditions are common and often interact with BPF in culturally informed ways. A cross-lagged panel analysis of 1608 Chinese adolescents revealed that the relationship between BPFs and depressive symptoms is bidirectional in boys but unidirectional in girls. Males exhibited reciprocal influences over time, whereas for females, the influence was unidirectional, with BPF predicting later depressive symptoms without a significant reverse effect (45). Meanwhile, the severity of BPFs and depressive symptoms was lower among boys than among girls (45). This divergence may reflect culturally ingrained gender norms, wherein girls are often socialized to internalize distress, potentially allowing BPF-related emotional dysregulation to more directly precipitate depressive states.

Trauma exposure and trauma-related disorders also demonstrate strong links with BPF in this population. Western epidemiological studies have reported Posttraumatic stress disorder (PTSD) comorbidity rates of 25-58% among BPD patients (46), and childhood adversity is commonly reported (47). Accordingly, a study in Chinese adolescents found that 64.9% of those high-BPF youth screened positive for dissociative symptoms (52.6%) and/or ICD-11 PTSD/CPTSD (41.2%). Trauma-related variables accounted for 52.9% of the variance in BPD features, reinforcing the notion that BPD may be meaningfully conceptualized as a trauma-related disorder in East Asian contexts (7). The substantial symptom overlap calls for careful differential diagnosis between BPD and PTSD. While both disorders share self-organization disturbances, PTSD/CPTSD results from prolonged trauma and alters threat perception, whereas BPD involves personality organization deficits that can develop through various paths—trauma included, but not the only path (48). Critically, abandonment fears and chronic emptiness are not CPTSD defining features; conversely, somatic dysregulation and trauma-specific flashbacks are not core BPD criteria.

BPD and Attention-deficit/hyperactivity disorder (ADHD) are frequently confused or co-occur in clinical practice, as BPF can be masked by overlapping impulsivity and emotional lability. However, BPD adolescents typically present with interpersonal difficulties and identity disturbance as primary complaints, whereas those with ADHD report attention problems and concentration deficits. BPD impulsivity is emotion-driven and interpersonal, with emotional dysregulation rapidly cycling around interpersonal triggers. In contrast, ADHD involves executive control deficits, and their lability reflects frustration intolerance when goals are blocked (49). BPD is also sometimes misdiagnosed as disruptive mood dysregulation disorder (DMDD) due to shared chronic irritability, whereas BPD mood storms are triggered by interpersonal cues and abandonment fears, and include core features in relationships, self-identity, and behaviors (49).

### 4.5 Neurological soft signs

Neurocognitive impairments, particularly in executive function and network connectivity, have been proven to be associated with childhood trauma and BPD symptoms (50, 51). Neurological soft signs (NSS)—subtle, non-localizing deficits in sensory integration, motor coordination, and complex motor sequencing—are significantly more prevalent in adolescents with BPF: 59.6% of BPF adolescents exhibited at least one NSS (vs. 34.8% of controls), and 42.7% exhibited two or more NSS (vs. 16.9% of controls) (52). Notably, sensory integration and disinhibition signs correlate positively with BPF severity, suggesting that neurodevelopmental vulnerabilities may contribute to the disordered self-regulation and behavioral control characteristic of BPD (52).

## 5 Psychotherapy and treatment: current status and challenges

The treatment of BPD adolescents is a challenging and critical focus in the field of mental health. Given the increasing prevalence of BPD and associated features among adolescents, the implementation of therapeutic interventions is particularly necessary. In the current clinical practice for BPD, medications are recommended only to alleviate acute symptoms of comorbid conditions due to their insufficient long-term efficacy (53). Relevant guidelines do not recommend medication as a core treatment approach (54). Instead, psychotherapy occupies a central position in BPD treatment (55). Treatment should rely on psychotherapy strategies rather than relying solely on medications. At present, research on the treatment of adolescents with BPD in East Asia is gradually increasing, but this field is still in its preliminary stages of development. Some therapies have been implemented in Western countries and shown convincing effects in treating patients with BPD. The most used approaches are Dialectical Behavior Therapy (DBT), Mentalization-Based Treatment (MBT), and Transference-Focused Psychotherapy (TFP), and DBT is the most widely used approach in the West. Similarly, in China, DBT is also regarded as the preferred treatment for BPD. This method has been applied across multiple hospital systems. Some studies in China confirmed the effect of DBT on symptoms such as emotional instability, self-harm and suicidal behaviors, depression, and anxiety in adults (56, 57). In contrast, some studies suggest that DBT demonstrates favorable short-term efficacy, but evidence for its long-term effectiveness remains insufficient (58), and its cross-cultural applicability requires further validation. Due to the increasing need for treating children and families, Dialectical Behavior Therapy for Adolescents (DBT-A) is gradually expanding through professional training. In clinical settings, DBT-A is implemented on a small scale. Multi-family group therapy grounded in DBT offers systematic intervention for adolescents with extreme emotional instability. It is a structured training and contains four core modules: mindfulness, emotion regulation, distress tolerance, and interpersonal effectiveness. MBT and TFP are also gaining attention. Research revealed some common elements across different therapies: stable therapeutic alliance, proper skills to manage emotional fluctuations, and adequate problem-solving approaches (59). An integrated treatment pathway is a potential option for BPD patients. However, high-quality evidence on the effect of these methods in East Asian adolescents is still limited. In Japan, a cross-sectional study (60) revealed the trend of psychotherapy methods for treating BPD in Japanese clinical settings. The most prevalent methods were supportive psychotherapy (52.3%), followed by psychoanalytic psychotherapy (40.5%) and cognitive behavioral therapy (37.3%), whereas only 11.8% and 8.5% of psychologists prefer DBT or MBT. Many factors may lead to this difference, including the early localization of psychoanalysis, the enduring influence of Jungian psychology, and indigenous approaches such as Morita and Naikan therapy (61, 62). Future studies should further examine both the efficacy and the cultural adaptation of these Western evidence-based approaches for adolescent BPD within East Asian contexts.

Some challenges exist in the clinical practice and intervention research for East Asian adolescents with BPD. First, it often occurs that adolescents’ BPD features are mistaken for adolescent “rebellion” or “mood swings”, especially when comorbid with depression and anxiety symptoms. Besides, East Asian emphasis on collectivism and social conformity, coupled with stigma surrounding mental illness, prevents some adolescents from receiving effective treatment. Moreover, due to the pathological personality traits that may impair their social skills, it is challenging to establish and maintain a stable therapeutic alliance with BPD populations. Therapists often need to place greater emphasis on set boundaries and face significant difficulties during treatment. Compared to adults, the specific features of adolescents place higher demands on therapists’ skills. However, treatment for East Asian adolescents with BPD is still in its early stages, with a relative shortage of professionals and gradually developing treatment concepts. Consequently, interventions may target the broad category of “adolescents exhibiting certain traits” rather than patients rigorously screened according to diagnostic criteria. More research is needed in the future alongside the development in this area.

## 6 Discussion

### 6.1 Cross-cultural comparison of assessment

On the aspect of measurement properties, some instruments, such as BPFS-C, have similar psychometrics across different cultures. However, East Asian studies on adolescents have relied almost exclusively on self-report measures, whereas Western clinical guidelines on adolescent BPD have been based on multi-informant assessment. Western methodology has drawn on both self-report measures and parent-report measures such as BPFS-C and BPFS-P (63), and semi-structured clinical interviews (e.g., CI-BPD, BPDSI-IV) (64, 65). This methodological difference partly reflects differences in sample sources. Community and school-based samples, which predominate in East Asian research, typically rely on self-report screening, whereas clinical samples rely more on diagnostic interviews and multi-informant assessments. The lack of validated East Asian versions of parent-report measures and semi-structured interviews remains a significant gap. Different informants have different perspectives on borderline symptoms. This matters because adolescents tend to underreport manifestations due to their limited insight into their own personality processes, which makes self-descriptions challenging (66). Informant-rated instruments are likely to observe behavioral symptoms that cannot be captured by self-report. Besides, diagnostic practices in this region also warrant consideration. In mainland China, the Chinese Classification of Mental Disorders (CCMD-III) does not include diagnostic criteria for personality disorders in adolescents, which may lead many clinicians to refrain from assigning BPD diagnoses to adolescents, frequently substituting these with classifications such as “childhood emotional disorder.” This diagnostic approach likely contributes to artificially reduced prevalence rates in clinical reporting. Structured and semi-structured interview tools based on the DSM-5 Alternative Model for Personality Disorders (AMPD), such as SCID-5-AMPD and STiP-5.1, have been recently developed and applied to adolescent populations in Western research (67). Although the Chinese versions of these tools remain in their preliminary stages of translation and adaptation, they represent an important direction for future assessment development.

These assessment gaps also have East Asian-specific clinical implications. Stigmatization of mental illness, collectivist values, as well as academic pressures, can impact symptom presentation and disclosure related to emotional dysregulation, self-harm, or interpersonal conflicts. Future research may consider (1): validating semi-structured interviews for East Asian adolescents to establish diagnostic gold standards, and (2) developing East Asian parent-report measures and other informant-rated instruments to enable multi-informant assessment. The comprehensiveness and accuracy of the assessment are thus improved.

### 6.2 Cross-cultural comparison of etiology

The East Asian adolescent findings on BPD etiology demonstrate a strong alignment with Western literature. Childhood trauma (68), emotional maltreatment (69), and peer victimization (70) have similarly been identified as risk factors in Western studies, with self-compassion and self-esteem (71) showing consistent inverse associations with BPD features. These convergent findings suggest that core etiological mechanisms for BPF are relatively stable across cultural contexts.

Discrepancies have surfaced in studies contrasting East Asian and Western research with regard to the relative predictive power of particular maltreatment subtypes for adolescent BPD. In Western research, a previous systematic review and meta-analysis suggested that childhood sexual abuse (CSA) was associated with roughly a five-fold increase in odds for a BPD diagnosis (72). Similar findings emerged from a recent comprehensive review that also identified CSA as a significant risk factor, with one longitudinal study indicating that adolescent girls who did not remit four years after the time of initial presentation were significantly more likely to have self-reported childhood sexual abuse (47, 73). By contrast, studies conducted within the East Asian context consistently found that although multiple subtypes of maltreatment are likely to contribute to the development of BPF, emotional abuse shows the strongest association with the development of BPD within this population (18, 33). The reasons why emotional abuse stands out in East Asian studies are not entirely clear. Cultural norms that discourage emotional expression may be one explanation. Emotional neglect tied to academic pressure is another possibility, while lower reporting of sexual abuse in East Asian samples cannot be ruled out either. When working with East Asian adolescents, emotionally invalidating environments deserve particular attention. Whether these East-West differences are real or just reflect how different studies measure and report abuse, the question is still unanswered. Direct cross-cultural comparisons using the same assessment methods would help clarify this.

Studies from both East Asian and Western contexts consistently point to similar parenting-related risk factors for BPD/BPF, including low parental warmth, coldness or rejection, and overprotection (23, 34, 74). East Asian studies have also identified anxious rearing as a significant correlate of BPF (34), a factor that has received less attention in Western literature. This finding likely reflects the East Asian socialization context—collectivism and intense academic expectations shape parenting in specific ways. Anxious rearing involves excessive parental worry, hypervigilance about the child’s performance, and transmission of parental anxiety. It may operate through several pathways: parents pass on their own occupational and economic stress, and communicate anxious expectations about the child’s future and the family’s welfare, which inadvertently convey that the child’s current state is inadequate, thus undermining emotional security and self-efficacy. These anxiety-driven parenting patterns warrant clinical attention: they may constitute a culturally specific form of family invalidation.

Both Eastern (13, 28) and Western (72, 75) research identifies low socioeconomic status as a risk factor for adolescent BPD/BPF, indicating that the effect holds across cultures. Family structure variables have been examined differently in each setting. East Asian research has reported significant roles of remarriage (28) and marital instability (13) in adolescent BPD development, while in Western literature, early maternal separation has been identified as a risk factor (76), but remarriage and marital instability have not been specifically studied in relation to adolescent BPD. In Chinese samples, non-singleton status and rural residence emerged as risk factors (13). Comparable Western data are largely absent. These discrepancies highlight how social policies and demographic contexts shape risk profiles differently across settings.

### 6.3 Cultural shaping of symptom presentation and underlying mechanisms

#### 6.3.1 Symptom expression in cultural context

Cultural factors may function not merely as contextual background but as structuring mechanisms that shape the expression of core BPD features. Paris (77) proposed that collectivistic societies contain social protective factors that suppress the overt manifestation of borderline pathology; BPD symptoms sometimes emerge after individuals migrate to individualistic settings, and lose communal structures that previously buffered trait-level vulnerabilities. Extending this framework, Najjarkakhaki and Ghane (78) suggested that migration may remove the protective layers that partially suppress impulsivity, emotional instability, and identity diffusion—core dimensions especially sensitive to sociocultural context. Ronningstam et al. (79) noted that East Asian self-restraint values attenuate outward impulsivity and redirect distress inward.

These cultural norms may directly shape how emotional regulation and interpersonal difficulties manifest. In East Asia, emotional dysregulation often manifests as expressive suppression, a culturally normative strategy that carries fewer psychological costs in collectivistic settings (80), yet this tendency is further exacerbated by academic pressure and anxious rearing that reinforce child inadequacy (34). In Confucian-influenced societies, the sense of self is embedded in familial and moral commitments—Ren (benevolence), Yi (righteousness), Xiu Yang (self-cultivation). Identity here involves one’s entire family, and shame follows when these family obligations are broken (81). This interdependent self-construal obscures the boundary between culturally normative relational dependency and pathological instability (78, 79). Consequently, fear of abandonment may appear as distress over disrupted relational harmony or loss of face (81). In such contexts, fear of being alone may itself be atypical, and interpersonal instability takes forms distinct from Western presentations (81). However, due to limited research, heterogeneous assessment tools, and a lack of direct cross-cultural comparisons, whether observed variations reflect genuine cultural differences or methodological limitations remains inconclusive. Further research employing standardized, multi-informant approaches is warranted.

#### 6.3.2 Theoretical frameworks and neurobiological evidence

Two established developmental frameworks help explain how culture may shape BPD pathology. Linehan’s biosocial model (35) posits that BPD arises from transactions between biological emotional vulnerability and invalidating environments. In East Asia, socialization practices such as emotional restraint and achievement-driven anxious rearing may represent culturally pervasive forms of invalidation (34). Childhood rejection, emotional neglect, and abuse contribute to rejection sensitivity, which in turn predicts BPD symptom severity (82). Unlike the “cold” or “rejecting” parenting often emphasized in Western studies, East Asian invalidation may operate through hypervigilance and achievement pressure rather than overt rejection. Interdependent self-construal may heighten vigilance to relational cues, turning routine social interaction into perceived rejection that feeds into idealization–devaluation cycles. The mentalization-based perspective (83) offers a complementary lens: BPD involves impairments in mentalization, the capacity to understand behavior in terms of underlying mental states. This capacity typically arises from disrupted early caregiving. Research has demonstrated that adolescents with BPD tend to hypermentalize—they make excessive and inaccurate assumptions about others’ mental states (84, 85). Meta-analytic evidence further indicates selective deficits in theory of mind reasoning and reflective function (86). The findings in East Asia align with the theory. Parenting that emphasizes obedience and emotional restraint may limit opportunities for developing reflective functioning and ultimately contribute to BPF (34). Meanwhile, mentalization deficits may also compromise interpersonal functioning. In East Asian contexts, where emotional expression tends to be indirect, such deficits can be particularly destabilizing.

Neurobiological evidence from East Asia suggests that brain alterations may serve as a neural link between culturally relevant risk factors and BPD pathology. Frontal-limbic circuits—including the prefrontal cortex, anterior cingulate cortex, amygdala, and hippocampus—are central to the disorder’s pathophysiology (49). Dysfunction in these regions may disrupt top-down regulation, leading to affective and behavioral instability. A recent systematic review of neuroimaging studies in adolescents with BPD revealed widespread alterations across three large-scale brain networks: the central executive network, the default mode network, and the affective network (87). In Chinese populations, studies by Chen and colleagues have identified these network-level abnormalities and further linked them to core BPD features, providing comprehensive neurobiological profiles in this population (88–90). Electroencephalography (EEG) studies offer complementary insights. A recent review highlighted that BPD individuals exhibit patterns of chronic hyperarousal and difficulties shifting between vigilance states, with EEG anomalies linked to childhood trauma, mentalization deficits, and dissociative symptoms (91). Similar findings have been observed in East Asian adolescents: A Korean EEG study linked childhood adversity and mentalization deficits to aberrant neural oscillations in BPD (92). Together, these findings suggest that culture shapes BPD across symptomatic and neural levels, embedding early social experiences into disorder-relevant circuits. This points to a bio-socio-cultural model, where core pathological features are conserved but neurobiological phenotypes show cultural specificity. Future cross-cultural studies are needed to test this hypothesis.

### 6.4 Future directions

#### 6.4.1 Cultural adaptation of treatments

Evidence-based treatments developed in Western individualistic contexts require systematic modification for East Asian populations, grounded in the region’s collectivistic values, family interdependence, and specific risk factors. Cross-cultural differences emerge in three aspects related to the adaptation of treatments. First, self-construal differs: interdependent self-definition may alter symptom presentation and treatment goals compared to independent self-definition (93). Second, the family system plays a central role, as family invalidation constitutes a core etiological factor in adolescents in East Asia. Third, emotion regulation patterns vary—greater reliance on expressive suppression in East Asian populations suggests standard Western strategies may be culturally mismatched (80). Across the therapeutic modalities, effective treatments should target emotional change, socio-cognitive change (including interpersonal patterns), and enhanced insight with modified defense mechanisms (94). Therapeutic alliance and therapist responsiveness operate as core mechanisms (94, 95). In East Asia, the therapeutic alliance must be reframed accordingly: rather than emphasizing individual autonomy, it should serve as a secure base for relational growth that respects family interdependence (93). Standard DBT strategies such as “opposite action” may require modification. Incorporating “skillful suppression” offers one example—a form of context-appropriate emotional containment aligned with social harmony. Therapist responsiveness similarly demands attunement to masked emotional storms, where intense internal experience meets restrained outward expression. Clinicians should attend to physiological cues and indirect communication rather than focusing solely on overt affect (93).

#### 6.4.2 Psychotherapy for individuals and intervention on family

As emotional maltreatment and invalidation are central etiological factors (18, 33), interventions should consider targeting emotional awareness and validation skills. Emotion regulation training is also essential, which is consistent with evidence that emotion dysregulation mediates the trauma-BPD relationship in East Asian samples (34). Self-compassion and self-esteem have been identified as significant protective factors in East Asian literature (18), which supports the inclusion of self-compassion-based intervention strategies in Western BPD treatments (96). This therapeutic component may align well with Buddhist-influenced cultural values in East Asia.

Anxious rearing is a culturally specific risk factor in East Asia (34). Family-focused interventions should consider addressing invalidation induced by anxiety-based parenting. These interventions should help parents manage their own anxiety and develop realistic expectations for their children. Validating the child’s intrinsic worth irrespective of academic achievement is a clinical priority. Specific techniques may include mindful parenting training to foster present-moment awareness and non-judgmental acceptance. Anxiety management skills are necessary to prevent the transmission of parental anxiety to children, and parent support groups can normalize difficulties and reduce isolation. The Chinese Invalidating Family Scale (44) provides a culturally appropriate assessment tool to identify targets for these family interventions. Addressing anxious rearing also requires broader societal changes. There should be policies reducing parenting pressure through educational reforms and de-emphasizing high-stakes testing. Workplace policies should support work-life balance, and social support systems are expected to address the economic insecurity of parents.

In addition, research has explored the incorporation of technological innovations and interdisciplinary approaches. This includes virtual reality (VR) to provide controlled settings for emotional exposure and mindfulness practice (97). Mobile health technologies have been developed for real-time emotion regulation and crisis intervention aids. Neuroimaging and physiological markers are recorded to track neurobiological responses to treatment. These approaches provide a foundation for achieving personalized and culturally adapted interventions.

#### 6.4.3 Enhancing school and community resources

Prevention and early intervention require cross-system cooperation. A comprehensive model can integrate mental health education in schools. It can implement early screening for high-risk adolescents, offer family support programs, and provide clear referral paths to community services. Risk factors unique to East Asia include rural residence and non-singleton status (13). These highlight the need to address structural inequalities in interventions. Adolescents in rural areas often have limited access to mental health care. In response to this need, telehealth-delivered interventions can be developed to reduce distance and cost barriers. Children left behind by migrant worker parents may face attachment disruption. Interventions should address the consequent caregiving gaps and train alternative caregivers. They can also facilitate structured contact maintenance between children and their parents. In the context of relaxed birth policies, adolescents from multi-child families may benefit from parenting programs that promote equal emotional attention across siblings.

Early identification and systematic management of precursor symptoms, including non-suicidal self-injury and emotional dysregulation, need particular attention. Future treatment research should move beyond single-therapy validation toward deeper exploration of therapeutic mechanisms, change processes, and patient-therapist-environment interactions. Through this iterative dialogue between theory and practice, the field can develop precise, effective, and humanized intervention pathways aligned with the developmental trajectories and cultural contexts of East Asian adolescents.
